# A chemical remediation technique for a nearly-total removal of arsenic and mercury from contaminated marine sediments

**DOI:** 10.1016/j.heliyon.2023.e22633

**Published:** 2023-11-19

**Authors:** Fabio D'Agostino, Antonio Bellante, Maria Bonsignore, Marianna Del Core, Laura Clarizia, Nadia Sabatino, Luigi Giaramita, Giorgio Tranchida, Salvatore Chiavarini, Mario Sprovieri

**Affiliations:** a Institute for Anthropic Impacts and Sustainability in the Marine Environment, National Research Council (IAS-CNR), via del Mare n. 3, 91021 Torretta Granitola (Trapani), Italy; b Department of Chemical, Materials and Production Engineering (DICMaPI), University of Naples Federico II, Piazzale V. Tecchio 80, Naples, 80125, Italy; c ENEA Casaccia Research Centre, Department for Sustainability, Via Anguillarese 301, Roma, 00123, Italy; d Institute of Marine Sciences, National Research Council of Italy (ISMAR-CNR), Arsenale-Tesa 104, Castello 2737/F, Venice, 30122, Italy

**Keywords:** Sulfides treatment, Sediment/soil washing for remediation, mercury and arsenic removal, Re-usage, Waste

## Abstract

After decades of industrial exploitation of the coast and consequent contamination of the sites and marine sediments, it became essential to recover the marine ecosystem by remediation methods to remove toxic contaminants. In this work, a remediation method was developed to clean marine sediments contaminated by arsenic (As) and mercury (Hg). The method can be applied to mobile platforms and is based on an environmentally friendly approach designed to minimise further contamination. The method was tested on two artificially contaminated sediments and two real samples collected from two highly contaminated sites in southern Italy, Augusta Bay and Bagnoli Gulf, characterised by high Hg and As concentrations, respectively. The method consists of four steps: washing with sodium hydroxide (NaOH) to remove metals associated with humic acid; Fenton-reaction using α-CycloDextrin (aCD) to stabilise Fe(II) at natural pH and oxidise As (III) and Hg (0 or I); complexation reaction with aCD; and complexation with sodium sulfide (Na_2_S) to remove Hg as soluble Hg-polysulfides. Compared to other remediation experiences in literature, this technique provides the best removal efficiency for As and Hg (ranging between 26 -71 % and 57–95 %, respectively). Considering the residual concentrations of As and Hg and the contamination threshold fixed by European Regulation for re-use, the treated sediment can be used in several civil and industrial contexts. The presented method operates in line with the principles of the circular economy to preserve natural resources, prevent secondary pollution, and promote the effective re-use of clean environmental matrices (soils, sediments and aqueous solutions), thus minimising landfill waste.

## Highlights

• Arsenic and mercury removal from clayey and sandy marine sediment with high contamination levels.
• Alkaline treatment to remove arsenic complexed with humic acid.
• Hyperoxidation and sulphide treatment to remove Hg.
• Solubilization of Hg as polysulphide species.
• Environmental sustainability of the treated marine sediment.

## Graphical abstract

**Figure:**
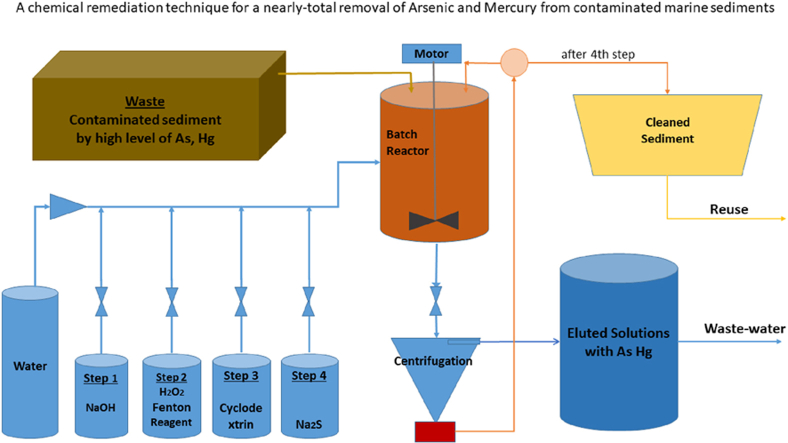

## 1 Introduction

Industrial activities, such as the production of construction materials and fertilisers, agriculture activities, forestry practices and improper urban development, have contributed to a gradual increase in heavy metal pollution worldwide. Nearly 2.8 million sites in Europe were characterised by polluting activities and still document relevant effects of environmental contamination, thus requiring suited actions for remediation/mitigation activities [1].

Heavy metal content in sediments is of serious concern since sediments may act as carriers and secondary sources for contaminants in the marine environment. Sediments are the preferential sink for trace metals in aquatic systems, wherein heavy metals are classified as follows: suspended forms, water-soluble species, colloids, and sedimentary phases ([[2], [3], [4], [5], [6], [7], [8], [9], [10], [11]]. Under specific physicochemical conditions, over 90 % of heavy metals released into an aquatic system may accumulate in sediments and distribute in the following diverse compartments: adsorbed on Fe/Mn oxyhydroxides or clays, complexed with organic substances, embedded into the lattice of primary minerals (e.g., silicates) or secondary minerals (e.g., carbonates or sulfates) or trapped inside amorphous materials [12,13]. Each form has a peculiar remobilisation capability defining its relative bioavailability and toxicity. Indeed, even a sudden change in physico-chemical conditions of the surrounding environment can lead to the release of the mobile heavy metals fractions from sediments to the aqueous medium. As a result, heavy metals become available to living organisms in aquatic systems. Some heavy metals exhibit remarkable toxicity to aquatic organisms and can affect human health by entering into food chain [14].

Mercury is a highly toxic and "possibly carcinogenic to humans" element [15]. Coal combustion, mining, and further industrial activities (e.g., Chlor-alkali plants and industrial/chemical wastewater discharge) account for the primary anthropogenic sources of Hg [[16], [17], [18]]. According to the Minamata Convention, the identification and characterisation of Hg-contaminated coastal areas are gaining increasing interest to assess potential risks for human health and the marine environment [19,20].

Arsenic is a carcinogenic and toxic metalloid. Pharmaceutical and pesticide production, past mining activities, leather manufacturing, and use of fossil fuels are among the main anthropogenic sources of As in polluted sites [21].

As part of the decision-making process aimed at selecting the most suitable remediation technologies applicable to a contaminated site, together with a profound exploration of the environmental and social context where the contaminant was found, it is essential to carry out an in-depth assessment/comparison of the different applicable technologies.

Remediation strategies for heavy metal-contaminated marine sediments include [22,23]: (i) isolation through in situ or ex-situ capping; (ii) in situ containment via solidification/stabilisation; (iii) sediment dredging followed by ex-situ techniques.

In situ remediation technologies are generally employed for marine sediments slightly contaminated with heavy metals. Nevertheless, such techniques exhibit low remediation efficiency on heavily polluted matrices [24]. In these cases, ex-situ strategies shall be considered the optimum remediation option. Most ex-situ remediation technologies developed for polluted soils can be employed for dredged marine sediments by considering their different physicochemical and microbiological characteristics [24].

Sediment washing, electrochemical treatment and biosorption are some of the most employed remediation technologies for heavy metals removal from dredged marine sediments [24,25]. However, most of these approaches have exhibited low Hg and As removal from polluted marine sediments. In addition, the removal of Hg, As, and other harmful contaminants through these approaches has exhibited unsatisfactory results when applied to fine-grained and low-permeable matrices [26].

Amongst physicochemical techniques, chemical separation attempts to mobilise these pollutants into a fluid phase where they can be concentrated, isolated, or degraded [27]. A solution made of an acid, a base, or a metal chelator may be employed as a leaching solution. Acid leaching is the preferential strategy to dissolve basic metal salts (i.e., hydroxides, oxides, and carbonates). Basic solutions are also employed for the release of selected metals adsorbed on mineral surfaces [27]. After metal dissolution, leached marine sediments require neutralisation, and the aqueous solution should be clarified to remove suspended particles. The extracted metal-rich solution should be adequately treated or concentrated by different possible technologies (i.e., ion exchange, precipitation in inert form, photocatalysis) [28]. The maximum removal efficiency of nearly 30 % for both As and Hg from marine sediments through chelator-assisted washing has been reported in the literature [29,30]. Chemical separation may also involve oxidation reactions by employing suited oxidising chemicals to transform sediment pollutants into less harmful products [31]. To this aim, sodium permanganate, sodium persulfate, ozone, and hydrogen peroxide are the most common oxidising agents adopted [32]. Such chemical species cause sudden and total removal of potentially toxic elements (PTEs) or partial degradation of other contaminants, eventually followed by complementary clean-up techniques [32]. However, the use of the above-mentioned oxidising agents is related to high operating costs or the production of impactful outcoming waste streams for which additional treatment techniques should be taken into account.

The present study aims to develop a novel ex-situ chemical oxidation technique to remove As and Hg from polluted marine sediments. Two artificially contaminated sediments and two real samples collected from highly contaminated sites in southern Italy (i.e., Augusta Bay and Bagnoli Gulf) are employed. The possibility of using sodium hydroxide to remove metals associated with humic acid, α-CycloDextrin to oxidise As(III) and Hg(0 or I), and sodium sulfide to remove Hg as soluble Hg-polysulfides is herein investigated for the first time through a sustainable and cost-effective multistep approach. Among the quantitative targets of this investigation, the achievement of removal efficiencies of As and Hg from polluted marine sediments significantly higher than those previously reported (i.e., 30 % for both As and Hg [29,30]) is pursued. The possibility of scaling up the proposed clean-up technology for real-scale implementation is also considered.

## 2 Materials and methods

The materials used in the remediation method were hydrogen peroxide 30 % (H_2_O_2_), sodium pyrophosphate (Na_4_P_2_O_7_), sodium hydroxide (NaOH) and α-cyclodextrin (aCD) reagent grade by Carlo Erba Reagent (Italy), and sodium sulfide hydrate extra pure (Na_2_S × H_2_O, 60–64 %; Thermo Scientific), used for the investigation test. During sample preparation and analyses, nitric acid (HNO_3_) supra-pure (Carlo Erba) was used.

### 2.1 Collection of samples

The remediation technique was applied to real polluted sediments and artificially contaminated sediment samples. Two areas were properly selected for the collection of real polluted sediments for their well-known contamination. Specifically, Augusta Bay (South-Eastern Sicily, N 37°12.244′, E 15°11.175′), named AUG, is a natural semi-enclosed marine area characterised by a severe state of Hg pollution caused by the activities of a chloralkali plant based on Hg-cell technology (1958–2003), which discharged of over 500 tons of Hg (as free elemental mercury (Hg0) and Hg complexes) directly into the sea at least until 1978, when the Italian legislation imposed restrictions [33,34].

The Bagnoli brownfield, named BAG, located in the Gulf of Pozzuoli (Napoli, southern Italy, N 40°48.691′, E 14°09.755′), suffered from the occurrence of a large number of industrial facilities, including the second largest Italian integrated steelwork plant (named ILVA) which exposed the marine sediments to a higher concentration of heavy metals and polycyclic aromatic hydrocarbons [[35], [36], [37]].

Two clean marine sediments from Capo Granitola Coast (western Sicily, N 37°33.895′, E 12°39.626′ and N 37°30.941′, E 12°33.263′), named CG_Sand and CG_Clay, were collected for their main grain size (similar to the real polluted sediments), to be artificially contaminated with Hg and As.

### 2.2 Sediments spiking

About 500 g of clean sediments (CG_Sand and CG_Clay) were artificially contaminated with 3 ml of 1000 mg/l of Hg(II) and 45 ml of 1.000 mg/l of As(V) standard solutions, respectively. After 2 h of homogenisation in a rotary vapour balloon, the sediment under vacuum was dried. Finally, this aliquot of 500 g of spiked sediment was mixed with another 2.5 kg of the same sediment and homogenised for 24 h in 10 L closed rotary tank bottle. These spiked sediments were named CG_Clay* and CG_Sand* and were frozen for over three months.

### 2.3 Sediments characterisation

Grain size and the heavy metals concentration were determined following the methods described below.

#### 2.3.1 Mercury determination in sediment and eluted solutions samples

Total mercury concentrations were measured by Direct Mercury Analyzer - atomic absorption spectrophotometer (DMA-80, Milestone s.r.l., Italy) following the US-EPA (7473) method. The analyses on solid samples (both soils and marine sediments) were run on about 20 mg of dry weight directly weighted in the sample holder and immediately analysed [38,39]. Otherwise, mercury on eluate samples was analysed by pouring 50 or 200 μl of liquid solution (depending on the expected concentration) directly into the sample holder.

#### 2.3.2 Heavy metals determination in sediment and eluted solutions samples

Heavy metals determination in sediment and eluted samples were carried out using ICP/MS by two different laboratories: the ICP/MS laboratory of CNR-IAS (National Council of Research, Institute of Anthropic Impacts and Sustainability in the marine environment (Italy, Tp) and by ENEA laboratory (Italy, Rm). The analyses were performed by previous microwave-assisted digestion with a mixture of concentrated hydro-chloridric and nitric acids [40,41] and then measured according to different EPA methods [42,43]. The acid mixture and sample were introduced into a closed Teflon vessel [44] and subjected to digestion using a DISCOVER SP-D80 microwave oven (CEM Corporation). Sample solutions and blank reagents for total dissolved metals were analysed by inductively coupled plasma with technologies in the Q cell (by Thermo Fischer - iCAP-Q).

#### 2.3.3 Grain size method

During the pre-treatment of the samples for the grain size analyses, any organic matter was removed by adding a solution of hydrogen peroxide (30 w/v – 100 vol%) and distilled water (1:4) and allowing it to react for 24–48 h. Then, deionised water was used for repeated cleaning to remove soluble salts, mainly sodium chloride and NaCl [45].

The grain size of the AUG and CG_Clay samples was determined using a Horiba Partica LA-950V2 laser particle size analyser. Data were subsequently processed using the HORIBA LA950 V. 5.20 software for Windows, which calculates statistical parameters and reconstructs the grain size distribution curves.

BAG and CG_Sand were analysed using a mechanical vibrating shaker and an ASTM sieve stack at half-phi intervals ɸ/2, with apertures ranging from 2 mm (-1ɸ) to 63 μm (4ɸ). The obtained data were computerised and elaborated with the "Fritsch Particle Sizer AUTOSIEB/A20″ software.

All of the samples were categorised according to their particle sizes using a logarithmic transformation (φ scale) of the geometric Wentworth classification [46], with limits expressed in ɸ (ɸ = -log2d, where d = diameter of grain in mm), according to the scale proposed by Krumbein [45].

#### 2.3.4 Quality control

Quality control on Hg, As, and other metals determination in sediment samples was carried out by analysing a Marine Sediment Certified Reference Material (PACS-3; NRCC, Canada) every ten samples to assess accuracy and precision (routinely) both for DMA-80 and ICP/MS instruments.

Referring to Hg concentration, determined in sediment using the DMA-80 instrument, accuracy and precision were estimated to be between 94 and 109 % and less than 7 % (RSD% on 5 replicates), respectively. Moreover, about 20 % of the total samples were analysed in duplicate to estimate the sediment samples' reproducibility (<7 %). The quality control on Hg in liquid samples (eluate) determined with DMA-80 was checked by analysing duplicate samples (about 20 % of the samples), and the reproducibility of the method was <15 %, the accuracy, assessed spiking Hg standard solution directly on samples, ranged between 85 and 120 %.

Referring to As, Cd, Co, Cr, Cu, Fe, Mn, Ni, Pb, V, and Zn concentrations, determined in sediment using ICP/MS, was carried out using PACS-3. The accuracy and precision within 94–106 % and greater than 7 % (RDS%, on 3 replicates), respectively, were estimated. Furthermore, duplicate samples (about 20 % of the total samples) were analysed to estimate reproducibility (about 94 %).

### 2.4 Remediation method

The developed remediation treatment was carried out using the magnetic agitator at four positions and has complied with soil/sediment washing based on reagents used in ISCO (in situ chemical oxidation) and complexation techniques. It was designed to remove the most significant amount of total As and Hg in sediment samples. Furthermore, this method was designed by looking at the results found in a mercury selective extractor procedure (SEP) in the sediments of Augusta Bay [47], where it was reported that the Hg in Augusta sediment is present as the sum of Hg(0) with Hg(I) and Hg(II), with a relative concentration, referred to the total Hg (THg), of about 80–95 % and 4–19 %, respectively. The presence of Hg(0) and Hg(I) should be attributed to the free elemental Hg coming from the Chloro-Alkali plant and Hg(II) to HgS [48].

The principle of this remediation method was: i) the use of pH solution always greater than 7 to preserve the carbonate minerals and ii) the use of a ratio sediment weight and volume solutions among 1:1 to 1:2 to minimise the treatment of the eluted solutions that need to be purified before the discharge.

Distilled and saltwater wash tests were performed on the spiked samples to verify the stability of As and Hg contamination. These tests showed no As and Hg elution, confirming their complexation with humic and sulfur substances already occurring in sediments.

The developed method consists of four main steps treating about 25 g of marine sediments (AUG, BAG, CG_Clay and CG_Sand) with 25 ml of the reagent solutions. After the reaction times, the two phases were separated using a centrifuge at 4500 rpm for 10 min. The solution was collected in a new test tube to determine Hg and As concentrations, and the sediment was resuspended using a vortex agitator with the same reagent solution and then re-agitated with the magnetic stirrer. This cycle was repeated three times.

**First step** - Humic acid separation - 25 g of sediment samples were treated overnight with 25 ml of a NaOH 1 M and Na_4_P_2_O_7_ 0.5 M solution to extract all soluble elements at pH 14 and humic acids from sediments [49]. Then, the sediment was separated from the solution by centrifugation at 4500 rpm per 10 min and washed twice with NaOH 1 M for about 2 min with a vortex agitator. These solutions were collected together and analysed to measure As and Hg concentrations. This step was developed to remove possible heavy metals linked with humic acid and also because it consumes a significant amount of H_2_O_2_ needed for Hg oxidation in the next step.

**Second Step** – Oxidation treatment – the sediment samples obtained by the previous step were treated with a modified Fenton reaction using the Fe(II/III) already occurring in sediment as an activator and α-cyclodextrin to stabilise it at natural pH (about 7.5). This reaction was carried out by mixing the sediment with 25 ml of distilled water with α-cyclodextrin (aCD) 0.1 M, and adding 2 ml of hydrogen peroxide (H_2_O_2_ 30 % v/v) every 30 min for a total time of about 3 h. After this reaction time, the solution was separated from the sediment by centrifugation and pooled in a separate test tube. The solutions were analysed singularly (about 25 ml per cycle) to determine the As and Hg concentrations. This step was developed for oxide As and Hg occurrence in sediments being species more soluble than in reduced form;

**Third step** – complexation with aCD - The sediment was resuspended and re-washed three times with 25 ml 0.1 M of aCD to remove Fe (and its complex compounds) and other possible heavy metals.

**Fourth step** – complexation treatment with Na_2_S – This remediation technique was developed using sodium sulfide as a complexant compound for Hg producing the soluble species Hg-polysulfides. The formation of mercury polysulfide complex (HgSx) in the presence of an abundance of sulfide and a negative redox potential (Eh < −200 mV) was previously reported [50]. The sediments from the previous treatment were agitated for 30 min. The solution was separated from the sediment using a centrifuge and pooled in a new test tube. Sediment was treated again with 25 ml of Na_2_S 1 M for 30 min. The three solutions were analysed singularly to determine the As and Hg concentrations.

## 3 Results and discussions

### 3.1 Grain size, pH, and Eh of sediments

The grain size characterisation of the sediments is shown in Table 1 as the result of 6 independent replicate tests. AUG and CG_Clay are constituted by silt and clay compounds (silt + clay is about 99.14 and 93.37 %, respectively), while CG_Sand and BAG are primarily composed of sand compounds (about 98.96 and 97.36 %, respectively). Before the grain size characterisation, pH and Eh were measured, and data were reported in Table 1.

**Table 1:** Grain size characterisation (as average ± standard deviation in percentage), pH and Eh.

Sample	Sand %	Silt %	Clay%	pH	Eh
CG_Clay	0.86 ± 0.17	42.28 ± 0.99	56.86 ± 0.91	7.35	−160 mV
CG_Sand	98.96 ± 0.05	1.04 ± 0.05		7.55	+96 mV
AUG	6.63 ± 0.40	57.43 ± 0.73	35.94 ± 0.76	7.45	−290mV
BAG	97.36 ± 0.22	2.64 ± 0.22		8.10	+6 mV

### 3.2 Heavy metals in sediment samples

Trace element concentrations were measured in ten aliquots of each sediment (AUG, BAG, CG_Sand, CG_Clay, CG_Sand* and CG_Clay*) before remediation treatments to determine the heavy metals' starting concentration level. The obtained data are reported in Table 2 as the average concentration ± standard deviation and described as follows.

i) AUG sediment compared with clean sediment samples, with similar texture (CG_Clay), shows similar trace element concentrations to those recorded in, except for Cr, Cu, Ni and Hg, which show higher concentrations (92.8, 45.2, 46.5 mg/kg, respectively). In particular, the Hg concentration (9.2 mg/kg) is over two orders of magnitude greater than CG_Clay. Cr, Ni and Hg concentrations also exceed the limits for a good environmental status required by Italian Regulation (D. Lgs 172/2015 – Table 2) for marine sediments. The high Hg concentration confirms its anthropogenic origin related to the activity of the chloro-alkali plant in Augusta Bay [ [19,33,34,[51], [52], [53], [54]]].
ii) BAG sediment samples, compared with clean sediment with similar texture (CG_Sand), show enrichment in Mn, As, Pb, Zn and Hg concentrations. Moreover, Pb concentration (133.6 mg/kg) exceeds the limits suggested by Italian Regulation (D.Lgs 172/2015 – Table 2). The average Mn, As, Pb, and Zn concentrations (996.3, 95.1, 133.6, 234.6 mg/kg, respectively) are higher than that found in Augusta (317.4, 17.4, 28.4, 92.4 mg/kg, respectively);
iii) CG_Clay compared with CG_Sand, CG_Clay shows heavy metals concentration enrichment one order of magnitude higher than values measured in CG_Sand per each metal (except for the Mn). These results are coherent with the greater metal affinity to the clay compounds with respect to the sand;
iv) as regards the spiked samples (CG_Clay* and CG_Sand*), the Hg level is coherent with the amount added to the clean sediment (about 1.2 and 1.1 mg/kg, respectively), while the As concentration is greater than the expected concentrations (36.7 and 31.8 mg/kg, respectively) because this element was already and naturally present in the sediments (Table 2). The other heavy metals were investigated to understand possible interaction or competition with Hg or As during the remediation treatment and to observe other possible metals' decontamination.

**Table 2:** Metals occurrence in dried sediment samples before the remediation treatment, average ± standard deviation expressed in mg/kg. Values in the bracket are the concentration of As and Hg before spiking.

	AUG	BAG	CG_Clay*	CG_Sand*	GES
As	17.4 ± 1.04	95.1 ± 8.32	36.7 ± 2.11 (20.4)	31.8 ± 2.25 (14.5)	12.00
Hg	9.2 ± 1.08	0.14 ± 0.01	1.2 ± 0.03 (0.044)	1.05 ± 0.07 (0.004)	0.30
Mn	317.4 ± 45.55	996.3 ± 87.04	263.1 ± 28.66	275.2 ± 20.41	
Cd	0.21 ± 0.03	0.29 ± 0.04	0.21 ± 0.03	0.08 ± 0.02	0.30
Co	17.2 ± 1.42	6.7 ± 0.57	9.8 ± 0.61	1.6 ± 0.08	
Cr	92.8 ± 14.21	19.8 ± 1.57	78.5 ± 6.95	9.1 ± 1.88	50.00
Cu	45.2 ± 2.02	11.2 ± 0.74	16.5 ± 0.92	1.1 ± 0.08	
Ni	46.5 ± 4.84	6.7 ± 0.72	27.1 ± 2.42	2.3 ± 0.33	
Pb	28.4 ± 1.67	133.6 ± 12.26	30.5 ± 1.99	3.9 ± 0.42	30.00
V	96.3 ± 5.36	92.4 ± 3.76	123.2 ± 9.29	15.2 ± 1.68	
Zn	92.4 ± 8.74	234.5 ± 16.86	79.6 ± 7.80	7.9 ± 0.60	
Fe^a^	3.7 ± 0.23	8.1 ± 0.67	3.5 ± 0.15	0.5 ± 0.07	

a values in percentage; GES: Good Environmental State.

### 3.3 As and Hg removed from marine sediments

Metal concentrations in the elute solutions obtained by each relative step and cycle of sediment treatment were reported in Table 3. The data represent the average value of three consecutive tests.

**Table 3:** As and Hg extracted from sediment per each treatment in micrograms (μg).

Treatment	AUG		BAG		CG_Sand*		CG_Clay*	
	As	Hg	As	Hg	As	Hg	As	Hg
1. NaOH	104.0	3.2	297.1	3.2	247.8	0.3	267.8	1.5
2. Oxydation (H_2_O_2_/Fe)	13.4	54.9	27.0	0.9	17.1		34.0	7.8
3. aCD complexant								
*aCD_I*	*5.2*	*15.3*	*7.0*	*0.5*	*2.5*	*0.1*	*9.9*	*1.7*
*aCD_II*	*1.9*	*4.7*	*7.0*	*n.d.*	*0.9*	*n.d.*	*6.9*	*0.4*
*aCD_III*	*1.5*	*2.6*	*4.1*	*n.d.*	*0.1*	*n.d.*	*5.2*	*n.d.*
aCD Total	8.6	22.6	18.1	0.5	3.5	0.1	22.0	2.1
4. Na_2_S complexant								
*Na*_*2*_*S_I*	*0.9*	*37.0*	*0.8*	*1.0*	*0.7*	*14.4*	*5.0*	*n.d.*
*Na*_*2*_*S_II*	*0.9*	*17.5*	*1.8*	*2.0*	*0.8*	*8.5*	*0.8*	*5.6*
*Na*_*2*_*S_III*	*0.4*	*10.1*	*1.2*	*0.2*	*0.3*	*6.3*	*0.5*	*4.9*
Na_2_S Total	2.2	64.6	3.8	3.2	1.8	29.2	6.3	10.5
Total extracted (ug)	128.2	145.3	346.0	7.8	270.2	29.6	330.1	21.9

n.d.: not detected.

**Fig. 1:**
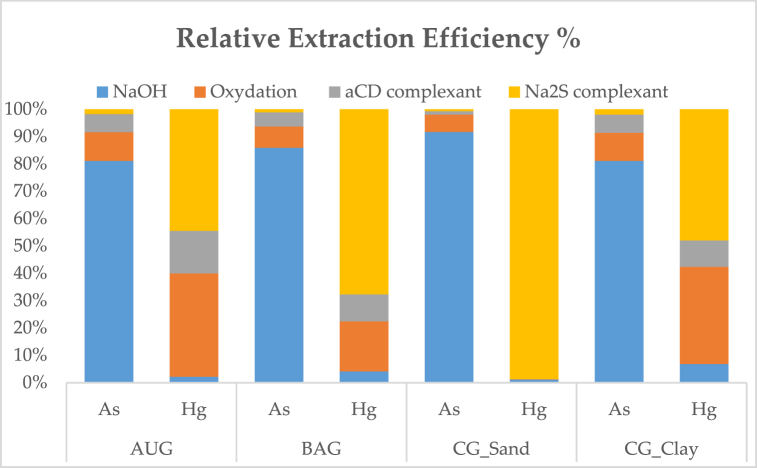
Relative Extraction efficiency % step by step of remediation method.

In the first step - treatment with NaOH 1 M − the removal efficiency is better for As than for Hg in all cases (Table 3 and Fig. 1). Specifically, the amounts of extracted As measured in about 25 ml of eluted solutions were 104.0, 297.1, 247.9, 267.8 μg in AUG, BAG, CG_Sand*, and CG_Clay*, respectively. The amounts of extracted Hg were 3.2, 3.2, 0.3, and 1.5 μg in AUG, BAG, CG_Sand*, and CG_Clay*, respectively.

During this alkaline process, humic acid was removed in all samples with high As concentrations, suggesting that As and humic substances form a soluble complex. An exception was found in the AUG sediment where the amount of As removal was half than of the other samples, probably because of the lower starting concentration. As regard BAG sediment, more and consecutive alkaline steps should be carry out to remove all As.

The second step - Fenton reaction modified – was developed to remove As and Hg by oxidation of As(III), Hg(0), Hg(I), or bound to organic compounds. The oxidation process was monitored by measuring the redox potential (Eh). The Eh values measured were less than −200 mV in each sample at the start and greater than +400 mV at the end of the process (after 3 h). After this step, the amounts of extracted.

As, measured in about 25 ml eluted solutions, were 13.41, 27.0, 17.1, and 34.0 μg in AUG, BAG, CG_Sand*, and CG_Clay*, respectively. The extracted Hg amounts were 54.9, 0.9, <0.1, and 7.8 μg in AUG, BAG, CG_Sand*, and CG_Clay*, respectively.

As theoretically supposed, a significant amount of Hg in sediments was in reduced form, probably as Hg(0) (Fig. 1). Previous studies found that Hg(0) is the predominant form of Hg in marine sediments [47]. Moreover, the amount of As removed by this process showed a low concentration of As(III) in sediments.

The third step – complexation with aCD - the As measured by the sum of each solution (of about 25 ml), were 8.6, 18.1, 3.5, 22.0 μg in AUG, BAG, CG_Sand*, CG_Clay*, respectively. The final amounts of extracted Hg were 22.6, 0.5, 0.1, and 2.1 μg in AUG, BAG, CG_Sand*, and CG_Clay*, respectively (Table 3).

As shown in Fig. 1, the sum of As and Hg eluted by the sediment was less than supposed, probably because the aCD did not form a hard complex with As and Hg.

The fourth step was developed essentially to remove the Hg as a poly-sulfide complex. After this step, the final amounts of the As extracted, measured in about 25 ml solution by the sum of each eluted solution, were 2.2, 3.8, 1.8, and 6.3 μg in AUG, BAG, CG_Sand*, and CG_Clay*, respectively, while the final amounts of the Hg extracted, in about 25 ml solution, were 64.6, 3.2, 29.2, and 10.5 μg in AUG, BAG, CG_Sand, and CG_Clay, respectively.

As theoretically supposed in this step only the Hg was removed and the greater amounts than the others step as shown in Table 3 and Fig. 1. So, due to anoxic condition, the sulfide compounds reacted with Hg in cinnabar form [55].

Finally, summing the amount of As and Hg extracted by each step (Table 3), the As total extracted was 128.2, 346.0, 270.2, and 330.1 μg in AUG, BAG, CG_Sand*, and CG_Clay*, respectively, and the Hg total extracted was 145.3, 7.8, 29.6, and 21.9 μg in AUG, BAG, CG_Sand*, and CG_Clay*, respectively.

Furthermore, the measured concentrations of As and Hg were converted into relative percentages extracted from each step (Table 3; Fig. 1) to highlight the relative efficiency of each extraction treatment. The following treatment showed a removal efficiency range of about.

1. NaOH: 81 ÷ 92 % and 1 ÷ 7 % for As and Hg, respectively;
2. H_2_O_2_/Fe Oxidation: 6 ÷ 10 % and 18 ÷ 38 % for As and Hg, respectively;
3. aCD complexation: 1 ÷ 7 % and 9 ÷ 15 % (and only 0.1 % in the CG_Sand*) for As and Hg, respectively;
4. Na_2_S complexation: 0.7 ÷ 2 % and 44 ÷ 98 % for As and Hg, respectively. A relevant exception was found for CG_Sand*, where the removal efficiency of Hg was about 98 %.

These results show that the most significant amount of the extracted As (81 ÷ 92 %) is obtained by the treatment with NaOH, probably because of its linkage with humic substances [56], or other soluble organic compounds. The low amounts of the extracted As (6 ÷ 10 %) at the oxidation step suggest that As(III) represents a low percentage of the total As occurrence in the sediment analysed. Moreover, a low amount of the extracted As (1 ÷ 7 %) was obtained at the step with the aCD complexant. Finally, the complete absence (0.7 ÷ 2 %) of extraction of As with Na_2_S demonstrates its insolubility in water.

The most significant amount of the extracted Hg (44 ÷ 68 %) was obtained by the complexation step with Na_2_S, confirming the affinity of Hg with sulfide compounds to form soluble Hg-polysulfide [50,57]. The percentage of 98 % found in GC_Sand* (spiked with Hg(II)) is probably due to the higher affinity specifically of Hg(II) with sulfides. The oxidation step also results in a significant amount of extracted Hg (18–38 %). A negligible amount was obtained in the treatment with NaOH and the complexation step, suggesting no links with humic acid and aCD.

### 3.4 Remediation efficiency

At the end of the extraction procedure, the treated sediment samples were dried and analysed to investigate the residual concentration of As, Hg and other heavy metals. Comparing the mean value before and after the remediation treatment, the amount of the metal removed was reported in percentage in Table 4. The developed remediation method showed the capacity to remove total As and Hg from sediments ranging from 26.4 to 71.2 % and 57.1–95.2 %, respectively. Refer to other metals, the method showed the removal of i) Co, Cu, V in the AUG sample of about 6.6, 41.8, and 5.2 %, respectively, ii) Mn, Co, Ni and Zn in the BAG sample of about 59.8, 16.7, 18.9 and 64.3 %, respectively, iii) Cu, Pb and V in the CG_Clay* sample of about 48.5, 15.1 and 40.7 %, respectively, iv) Cd and Zn in the CG_Sand* sample of about 15.0 and 8.2 %, respectively.

**Table 4:** Remediation efficiency percentage of As, Hg, and other metals.

	AUG %	BAG %	CG_Clay* %	CG_Sand* %
As	59.9	26.4	71.2	55.9
Hg	76.3	57.1	92.4	95.2
Mn	n.c.	59.8	n.c.	n.c.
Cd	n.c.	n.c	n.c.	15.0
Co	6.6	16.7	n.c.	n.c.
Cr	n.c.	n.c.	n.c.	n.c.
Cu	41.8	n.c.	48.5	n.c.
Ni	n.c.	18.9	n.c.	n.c.
Pb	n.c.	n.c.	15.1	n.c.
V	5.2	n.c.	40.7	n.c.
Zn	n.c.	64.3	n.c.	8.2

n.c.: no significant changes observed.

These results confirm the excellent performances of the reported chemical remediation technique, both in clay and sand sediment, to remove As and Hg from CG_Clay*, CG_Sand*, and AUG sediments. This remediation treatment shows low efficiency in removing As and Hg from BAG sediment, probably due to the high concentration of stable As and Fe. In fact, the amount of As extracted from BAG sediment is similar to the other sediment samples (see Table 3), so more time or more washing cycles with NaOH 1 M should be required to enhance the As removal. Probably, the high level of Fe occurrence in BAG sample and its high affinity with sulfide ions consume the more significant amounts of Na_2_S added and leave an insufficient sulfide concentration to form soluble Hg-polysulfide compounds. Thus, the sulfide amount should be increased in step 4 to improve the Hg remotion.

Furthermore, the pH and Eh measured at the end of the extraction procedure in all sediment samples were about 12 and –400 mV respectively. These values are due to the greater amount of sulfide than at the start of the procedure. To reduce the pH and increase the Eh, a washing step with water should be carried out to reduce the residual amount of sulfide in sediment samples.

## 4 Conclusion

The proposed remediation method proved its efficiency in removing a significant amount of As and Hg from contaminated sediments. The removal efficiency (ranging from 56 to 72 % and from 76 to 95 % for As and Hg, respectively) shows an improved removal capacity compared to previous literature results, which report maximum extraction percentages of As and Hg of about 30 % [29,30,36]. The BAG sample represents an exception, as it requires a separate specific treatment due to its peculiar physiochemical characteristics (abundance of As, Fe).

This method, developed on a laboratory scale, can also be up-scaled by using ordinary mechanical agitators to promote contact and reaction between the reagents, a hydro cyclone to separate the sand grain size from the eluted solution and clay, and a centrifuge to separate the clay from the eluted solution. In fact, all these apparatus and reagents are already adopted in soil/sediment washing plants, on in-situ and ISCO soil remediation techniques, providing reasonable support for the presented techniques. This remediation method can also be applied for soils due to the similarity between marine sediment and soil matrices.

The use of Na_2_S as a complexing agent for Hg demonstrates that stable forms of Hg or HgS (cinnabar) accumulated in marine sediment can be efficiently extracted, favouring their tendency to form a soluble Hg-polysulfide species and their mobility in the water column when the biogeochemical process leads to an increase of sulfurs concentrations. Based on the residual concentrations of As and Hg in the sediments and according to the European Regulation, the sediments can be re-used for civil and industrial applications coherently with sustainability criteria.

Considering these results, another work is in progress to evaluate the up-scaling of this remediation method, using a soil washing plant with a treatment capacity of 1 ton of sediment per hour and also looking at the economic feasibility.

## Funding

This research has been conducted within the framework of the Marine Hazard project 416(PON03PE_00203_1). In Italian language: *Ministero dell’Istruzione, dell’Università e della Ricerca (MIUR) – PON “Ricerca & Competitività” 2007–2013 – Decreto Direttoriale 713 Ric. Del 29 ottobre 2010 – Titolo III Creazione di nuovi distretti e/o Aggregazioni Pubblico-Private.*

## Additional information

No additional information is available for this paper.

## CRediT authorship contribution statement

**Fabio D'Agostino:** Conceptualization, Data curation, Investigation, Methodology, Project administration, Resources, Validation, Writing – original draft. **Antonio Bellante:** Investigation, Methodology, Writing – review & editing. **Maria Bonsignore:** Investigation, Validation. **Marianna Del Core:** Investigation, Validation. **Laura Clarizia:** Project administration, Writing – review & editing. **Nadia Sabatino:** Investigation, Resources, Validation. **Luigi Giaramita:** Investigation, Validation. **Giorgio Tranchida:** Investigation, Validation. **Salvatore Chiavarini:** Investigation, Resources, Validation. **Mario Sprovieri:** Conceptualization, Funding acquisition, Project administration.

## Declaration of competing interest

The authors declare that they have no known competing financial interests or personal relationships that could have appeared to influence the work reported in this paper.
